# Bone Marrow NK Cells: Origin, Distinctive Features, and Requirements for Tissue Localization

**DOI:** 10.3389/fimmu.2019.01569

**Published:** 2019-07-10

**Authors:** Valentina Bonanni, Giuseppe Sciumè, Angela Santoni, Giovanni Bernardini

**Affiliations:** ^1^Department of Molecular Medicine, Sapienza University of Rome, Laboratory Affiliated to Institute Pasteur-Italia, Rome, Italy; ^2^IRCCS, Neuromed, Isernia, Italy

**Keywords:** natural killer cells, bone marrow (bm), infection–immunology, innate lymphoid cell, chemokine receptors

## Abstract

NK cell maturation is a continuous process, which initiates in the bone marrow and proceeds in peripheral tissues, where NK cells follow distinct differentiation routes. Drastic phenotypic changes are observed during progression from precursors to mature NK cells, including changes of expression and functionalities of several chemoattractant receptors. Upon differentiation, mature NK cells migrate outside the bone marrow; as well, peculiar subsets of NK cells can also home back to or localize in this anatomic compartment to play specific functions. In humans, NK cells with a tissue resident phenotype have been identified in bone marrow, sharing similarities with tissue resident memory CD8^+^ T cells; while in mouse, long-lived NK cells undergo homeostatic proliferation in this site during viral infections. The mechanisms underlying NK cell subset localization in the bone marrow have only recently started to be investigated, especially in pathological settings such as tumors or infections. In this review, we discuss the phenotype and function of NK cells as well as their requirements for bone marrow maintenance and/or homing.

## Introduction

Natural Killer (NK) cells are innate lymphocytes able to recognize and kill cancer or virus-infected cells (1). They account for 5–20% of the mononuclear cells of the peripheral blood and the spleen. They also produce cytokines, among which interferon (IFN)-γ delivers signals to the innate component of the immune system, which activate the inflammatory process in defense of the organism. Activation of NK cell function following interaction with a target cell is the result of the integration of signals generated by inhibitory and activating receptors expressed simultaneously by NK cells and engaged by the ligands present on the target cells (2). By acting early during cell infection or transformation, before and independently of specific immunity, they take part to the first line of the immune response. These characteristics make them fundamental as a defense mechanism.

Recently NK cells have been re-categorized as part of the innate lymphoid cells (ILCs). ILCs have been characterized in three groups. The group 1 comprises cells expressing the transcription factors T-BET and producing the T helper cell type 1 (Th1)-associated cytokine IFN-γ, including NK cells (3).

Conventional NK cells appear to be the only cytotoxic cells, while all the other ILCs follow the pattern of helper CD4 T cells and produce cytokines and other soluble factors that help adaptive immune response development.

The transcription factor EOMES is expressed by NK cells but not ILC1, thus allowing to distinguish this two subsets (4). Mouse and human ILC differentiation process proceeds gradually from hematopoietic stem cells (HSC) to the precursor of the lymphoid line, including the common lymphoid progenitor (CLP) in the mouse and the CLP-like hematopoietic progenitor cells (HPC) in humans (4).

The bone marrow (BM) is considered the main site for ILC differentiation (also termed ILC-poiesis) in the adult, containing a wide spectrum of progenitors and precursors able to give rise to cells having different degrees of multipotency, commitment, and maturation (4). Several multipotent ILC precursors have been defined in mice, including the α-lymphoid progenitor, early innate lymphoid progenitor, common helper innate lymphoid progenitor and innate lymphoid cell progenitor (5). While single-cell RNA-seq approach has been helpful to unravel the complexity of these precursors by improving the definition of markers and transcription factors associated with the ILC fates (6), their pluripotency has been continuously redefined by using different mouse models (7–9).

ILC precursors express several chemotactic receptors and molecules associated with tissue homing, including CXCR5 and CXCR6 (10). In particular, the expression of CXCR6 in these cells has been related to egress from BM, as supported by evidence in *Cxcr6*^−/−^ mice showing accumulation of ILC precursors in this organ (11). Remarkably, these mice have normal numbers of peripheral ILCs, due to *in vivo* mechanisms compensating *Cxcr6* deficiency that include increased proliferation of tissue resident cells (11). Recently, a peculiar ILC precursor has been described in human peripheral blood, which can give rise to mature cytotoxic NK cells and ILC subsets (12). These findings, along with the presence of cells having progenitor phenotypes in several peripheral tissues, imply the existence of homeostatic mechanisms of BM egress for ILC precursors. Thus, one of the paradigms in the ILC field is based on the capacity of ILC precursors to leave the BM and complete their differentiation programs in the tissues. This behavior mainly discriminates helper ILCs, which develop *in situ*, from NK cells, able to recirculate, and has been corroborated by findings obtained from parabiosis experiments, in mice (13, 14).

NK cells develop from a multipotent progenitor, the HSC, in a continuous differentiation process encompassing several stages, characterized by modulation of multiple cell surface markers (15). Developing cells acquire the expression of the IL-15 receptor (IL-15R), including the common β chain of the IL-2 and IL-15 (CD122) (16). The acquisition of the CD122 represents an important step in the NK cell differentiation since IL-15 promotes NK cell differentiation, maturation, and survival and is constitutively produced by BM stromal cells and can be induced in monocytes and dendritic cells *in vivo* (17, 18).

NK cell differentiation and maturation have been traditionally thought to occur exclusively in the bone marrow (BM), but evidence in humans and mice suggests that precursor and immature NK cells can also migrate in secondary lymphoid tissues (SLT) to complete maturation (19). Human NK cells develop from hematopoietic stem cells (HSCs) and during transition from CD56^high^ into CD56^low^, they undergo a progressive loss of NKG2A and expression of KIRs, CD57, and NKG2C on terminally differentiated NK cells (20–22). Moreover, a new Lin^−^CD34^+^DNAM-1^bright^CXCR4^+^ CLP precursor has been found in the peripheral blood of patients with chronic inflammatory conditions. The phenotype of these cells suggests that they originate from the BM as they still retain the CXCR4 and DNAM-1 receptors, and that they are released from endosteal niches due to bone remodeling occurring during chronic inflammation (23).

Mouse NK cells develop from HSCs encompassing four developmentally related subsets that can be distinguished based on expression levels of the integrin chain CD11b and of a member of the TNF receptor superfamily, CD27.

The bone marrow is not only a place for development and maturation, but BM NK cells perform important functions for defense against infections and tumors linked to their ability to traffick and/or reside in this organ (24–28).

## NK Cells and Other ILC Populations in the Bone Marrow

Several members of the chemokine family influence NK/ILC tissue localization by regulating their release from the BM as well as their tissue homing and retention. Beside this type of conventional NK cells that can be found in circulation, tissue resident NK cells present specific characteristics that involve for example CD69, possibly linked to suppression of sphingosine 1-phosphate receptor-1 expression which retains immune cells in lymph nodes and tissues. Another mechanism is the engagement of chemokine receptors, like for example CXCR6 and CCR5, that are highly expressed on tissue-resident NK cells in human lymphoid tissues and liver, while peripheral blood-derived NK cells can be recognized by expression of CCR7 (29).

NK cell subsets display a differential pattern of chemokine receptor expression. In humans, CD56^high^ NK cells are targeted to lymph nodes *via* CCR7, preferentially express CXCR3 and have higher CXCR4 expression levels as compared with CD56^low^ cells. CXCR1, ChemR23, and CX_3_CR1 are expressed only by CD56^low^ NK cells. ILC subsets have differential tissue tropism, reflecting their transcriptional and functional states. Transcriptomic analyses established in the context of the Immgen project have revealed both specific and overlapping expression patterns for chemokine receptors in mouse ILCs (30). CXCR3 is one of the chemokine receptors showing subset specificity. This receptor is typically associated to the type 1 response and in general with T-BET expressing ILCs, including NK cells, ILC1 and a subset of ILC3 expressing NCRs. The chemokine receptors CCR4 and CCR8 are associated, instead, with the type 2 response and are specifically expressed on ILC2. Finally, CCR6 and CXCR5 are found mainly on lymphoid tissue inducer (LTi)-like cells (30–33). Examples of chemokine receptors widely express on ILCs include CXCR4 and CXCR6.

Upon maturation, mouse NK cells start to express sphingosine 1-phosphate receptor (S1P5), and co-expression of KLRG1 and the chemokine receptor CX_3_CR1 identifies a late maturation stage with unique functional properties (34). Mature populations of NK cells accumulate in the BM of CX_3_CR1 and S1P5-deficient mice, and display defective translocation from the BM parenchyma to the vasculature indicating that these receptors contribute to the egress of specific NK cell populations from the BM (35, 36). Even though immature CD11b^low^ NK cells represent the predominant population in BM, they are poorly mobilized into circulation due to CXCR4 mediated retention (37).

Besides immature NK cell populations, BM comprises both potentially cytotoxic NK cells, trafficking from the blood, and stably resident cells expressing specific markers of tissue retention (CD69) and chemokine receptors (CCR5, CXCR6). Indeed, a third distinct CXCR6+CD69+ subset of NK cells populating lymphoid tissues, distinct from the conventional CD56^high^ and CD56^low^ NK cells, represents a relevant fraction of human BM NK cells and displays lower functionality, possibly linked to organ-specific immunomodulatory functions (38). Compared to classical NK cells, the BM resident NK (BMrNK) cells display lower proliferative capacity, cytolytic granule content, and expression of KIRs and DNAM1, while they express higher TIGIT levels. Interestingly, this population does not express molecules implicated in localization of resident cells in other tissues such as the adhesion molecules CD49a and CD103 (39, 40).

The role of CXCR6 in promoting BM colonization by these cells was not investigated, but a fraction of immature BM NK cells was found to express CXCR6 also in the mouse, and deletion of *Cxcr6* gene limits the egress of this population into the blood circulation. Similarly to CXCR3 and S1PR5, expression of this chemokine receptor is regulated by the transcription factor TBET since it is suppressed in BM NK cells of TBET knockout mice (41, 42).

Mature ILCs are not usually found in the BM; however, as for NK cells, this site contains a peculiar subset of ILC2 having an immature phenotype. These cells differ from terminally differentiated ILC2 for the lack of the chemokine receptors CCR4 and CCR8, as well as KLRG1 expression (43). Currently, our knowledge on the mechanisms underlying ILC2 egress from BM remains limited. A role for IL-33 in this process has been proposed based on the observation that mice deficient for *Il33* or *St2* show a drastic reduction of ILC2 in the tissues. This is in contrast with the accumulation of these cells observed in the BM. Indeed, when the IL-33/IL-33R axis is disrupted the ILC2 present in the BM shift the expression of chemokine receptors showing increase of CXCR4, which results in increased retention in the BM (44).

Recently, the concept of ILC2 strictly seen as tissue-resident cells has been revised based on their ability to traffick upon activation (45). This paradigm shift, at least for ILC2, is based on the effects of IL-25 administration *in vivo* or helminth infection in mice (45, 46). Indeed, lung localization of inflammatory ILC2 minimally involves the recruitment of ILC2 from the BM. Conversely, the intestine is the reservoir where these cells originate and come from. Remarkably, the lung inflammatory ILC2 generated in the intestine keep a distinct transcriptional profile from the lung-resident ILC2. This interorgan trafficking of inflammatory ILC2 relies on S1P.

## BM NK Cells in Immune Responses Against Infections

Beside its role in supporting ILC-poiesis, the BM contains a significant proportion of mature lymphocytes, including NK cells which can participate to the immune response *in situ* or be mobilized into blood to migrate to peripheral tissues during systemic or local microbial infection.

## BM NK Cells and Viral (MCMV, RSV, Influenza) Infection

**Figure 1:**
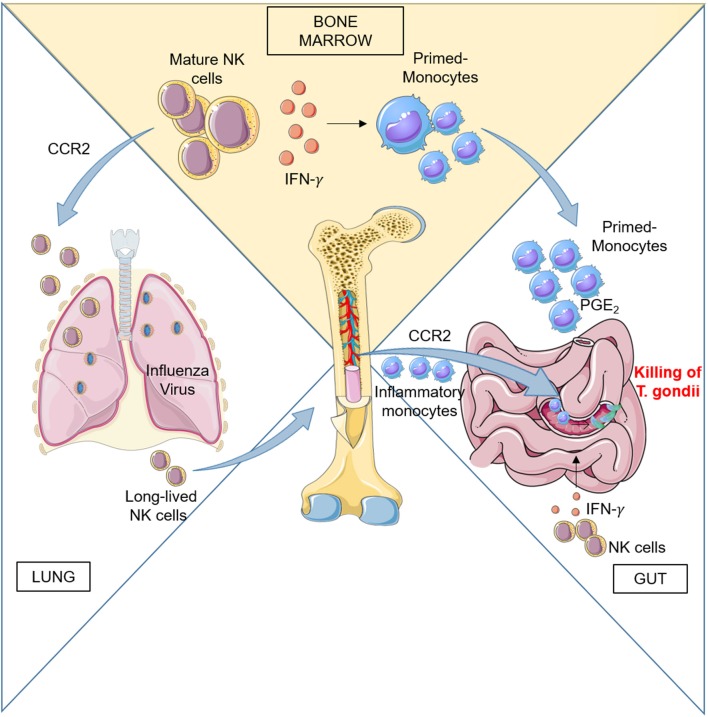
Left: CCR2 regulates BM NK cell recruitment to the lung during influenza virus infection. During infection, activated NK cells home back to BM and persist there to provide a pool of effector cells which proliferate quickly upon subsequent infections. Right: Peripheral NK cells participate to the activation of the immune response during Toxoplasma gondii infection by promoting differentiation of inflammatory macrophages and monocyte-derived DC. Ileitis promoted by inflammation can result in severe tissue damage, but recruitment in the gut of PGE2- and IL-10-producing monocytes generated in BM under the influence of NK cells dampens exaggerated inflammation and ensures epithelial barrier protection.

Early studies in mice indicated that systemic type I IFN induction by poly(I:C) treatment, LCMV and MCMV infections elicit NK cell responses (47). These results suggested that one systemic IFN-α/β induction is required to activate blast NK cell precursors located in BM and drive their efflux to peripheral compartments leading to increased NK cell cytotoxic activity and appearance of blast NK cells in the spleen. The mechanisms of BM NK cell mobilization have not been investigated but likely involved regulation of chemokine receptor function since, *Ccl2*- and *Ccr2*-deficient mice showed reduced proportions of NK cells in the liver during MCMV infection (48). The role of CCR2 in BM NK cell response to viral infection is also supported by evidence obtained in a mouse model of respiratory virus infection: NK cells migrated from the BM to the airways of mice and this process was attributed to CCR2-mediated egress from the BM using mixed-BM chimera mice studies (49). The author demonstrated that upon influenza virus infection, a proportion of *Ccr2*^−/−^ significantly lower than WT NK cells was recovered from the bronchoalveolar lavage of infected mice and corresponded to a mild increase of *Ccr2*^−/−^ NK cells in the BM (Figure 1).

In addition to recruitment in infected tissues, peripheral immature and mature NK cells also home to the BM during influenza infection and can respond to subsequent viral challenge by proliferating there (50). Using an adoptive transfer model, van Helden et al. demonstrated that during a first infection cycle, the BM contained not only immature NK cells but also mature, long-lived NK cells that had migrated back from the periphery to undergo both homeostatic and infection-induced proliferation.

Why do NK cells proliferate in BM upon influenza virus infection? The authors proposed that the BM constitutes a site for maintenance of NK cell immunological memory, a function already documented for plasma cells and memory T cells (51, 52). Homeostatic NK cell proliferation mediated by key cytokines expressed in BM may thus be responsible for the preservation of long-lived NK cells in the absence of viral stimuli; as for NK cells preactivated with cytokines *in vitro*, these NK cells are not pathogen-specific as they responded to the unrelated respiratory syncytial virus similar to influenza virus (50).

Other evidence suggests the existence of a pre-existing pool of BM NK cells with elevated effector capacity: upon systemic viral-like stimulation NK cells display elevated motility in the BM cavities possibly representing a search for accessory cells to interact with and target cells to kill, two processes critical to activate NK cell function and for anti-viral response (53). This response was associated with upregulation of effector molecules, followed within 24 h by induction of genes required for cell cycle and DNA replication. BM NK cell response paralleled that of spleen NK cells, although the formers were faster to proliferate. Using two-photon microscopy, NK cells have been observed leaving the blood sinusoids and displaying distinctive features of strong activation in the BM parenchyma. In this compartment, NK cells increased size and motility and underwent multiple interactions with CD11c^+^ cells and cell division.

The mechanisms of NK cell migration and/or proliferation in BM in response to virus infections, however, remain unknown. Furthermore, it is not clear if BM NK cells that undergo extensive proliferation during infection in peripheral organs include subpopulations able to perform specific functions *in situ*.

## BM NK Cells in Mouse Models of *Toxoplasma gondii* Infection

BM NK cell activity during early infection could also impact the myeloid cell compartment *in situ* during *Toxoplasma gondii* (*T. gondii*) infection. *T. gondii* is a protozoan parasite that infects intestinal enterocytes and spreads into the submucosa. Upon infection, inflammatory monocytes exit the BM and home to the lamina propria where they differentiate into TNF-α/inducible nitric oxide synthase (iNOS)-producing (Tip)-DC that control infection. Inflammatory monocytes failed to exit the BM in *Ccr2*^−/−^ mice, although their number was upregulated following infection indicating that CCR2 is critically involved in the egress of Tip-DC precursors from the BM to the blood, similarly to previous observation in *L. monocytogenes*-infected mice (54–57).

By using depleting antibodies, NK cells were initially shown to be essential for early parasite control (58). Subsequent studies showed that NK cell-produced IFNγ is a dominant, early protective mechanism, possibly acting directly on infected cells or stimulating a cytotoxic T cell response (59, 60). More recent studies performed in a peritoneal infection model, have demonstrated that IFNγ acts both by promoting tissue-recruited monocyte differentiation into IL-12 producing DC at site of infection and the loss of resident mononuclear cell population which are not able to control infection.

Evidence obtained in these and other studies led to the conclusion that inflammatory Ly6C^hi^ monocytes acquire appropriate functions after entry into infected tissues and in response to local signals (57, 61, 62).

Although oral *T. gondii* infection leads to a localized response that resolves without severe pathology, oral infection of certain strains of mice leads to epithelial damage and gut pathology driven by an aggressive type 1 immune response to commensal bacteria. However, despite severe ileitis, *T. gondii*–infected mice can survive the infectious challenge due to acquisition of a PGE2-dependent regulatory function acquired by Ly6C^hi^ inflammatory monocytes (63). Recently Askenase et al. showed that mature BM NK cells are responsible for the acquisition of this regulatory function by interacting with differentiating monocytes before their release into the circulation (26) (Figure 1). NK cells in BM were activated by signals derived from infected tissues in the periphery. Acquisition of regulatory function was associated with a MHCII^+^Sca-1^+^CX3CR1^−^ phenotype by Ly6C^hi^ circulating monocytes and was a common response to several type of infections, in addition to *T. Gondii*. The authors evidenced that IFNγ was responsible for a dramatic alteration of the transcriptional program of monocyte progenitors in the BM early during infection and before terminal differentiation and egress. BM NK cells were initially the only population producing IFNγ in the BM, with minimal contribution by T cells or type 1 ILCs, while IFNγ production was also observed in T cells at later time. The results demonstrated that IFNγ dependent mechanism of regulation can be designed to prepare monocytes for recruitment to barrier tissues and prevent immunopathology and tissue damage thanks to their capacity to produce PGE2 and IL-10.

Overall, NK cells and probably other leukocytes do not only act as effector cells in the periphery but also regulate myeloid cell differentiation in the BM, thus shaping the immune response during infection.

## Conclusions

Correct localization of NK cells in BM have a fundamental role in several aspects of NK cell-mediated immune response *in vivo*. BM can represent a location suitable for self-renewal and persistence of NK cell population with enhanced functional capacity. This is of clinical importance for the reconstitution of immune compartments during viral infections. It would be of great importance to understand if a similar expansion of memory-like populations could be observed during cancer growth, since experienced BM NK cells of cancer patients may be potentially able to mediating continuous surveillance against the recurrence of cancer. On the other hand, it is not known if the existence of NK cells capable of modifying the function of monocytes in BM applies to cancer growth outside the BM since it may contribute to generation of monocyte with immune suppressive functions.

The origin and phenotype of the BM NK cells promoting this function is not clear. In particular, it has not been investigated whether these NK cells reside stably in the BM, occupying stable niches or whether they migrate there participating to the systemic immune responses similarly to virus infections. In this regard, it would be interesting to clarify if the phenotype of these cells overlaps with that of tissue resident NK cells identified in BM since BrNK cells have enhanced IFNγ production capacity and low killing potential, thus suggesting that they can shape hematopoiesis under specific conditions.

## Author Contributions

VB wrote the introduction section and contributed to the paragraph concerning NK cells in BM. GS wrote the sections regarding innate lymphoid cells contributing to the introduction section and the paragraph concerning the regulation of ILC distribution in bone marrow. AS contributed to paper writing and revised the paper. GB revised the literature and wrote the paragraphs concerning NK cells in BM and BM NK cells in infection.

### Conflict of Interest Statement

The authors declare that the research was conducted in the absence of any commercial or financial relationships that could be construed as a potential conflict of interest.
